# IRTKS Promotes Insulin Signaling Transduction through Inhibiting SHIP2 Phosphatase Activity

**DOI:** 10.3390/ijms20112834

**Published:** 2019-06-11

**Authors:** Chongchao Wu, Xiaofang Cui, Liyu Huang, Xueying Shang, Binghao Wu, Na Wang, Kunyan He, Zeguang Han

**Affiliations:** Key Laboratory of Systems Biomedicine (Ministry of Education), Shanghai Center for Systems Biomedicine, Shanghai Jiao Tong University, Shanghai 200240, China; acamas@sjtu.edu.cn (C.W.); yueyue08@sjtu.edu.cn (X.C.); huangly@sjtu.edu.cn (L.H.); shangxueying2016@sjtu.edu.cn (X.S.); haowbdreamer@gmail.com (B.W.); nawang@sjtu.edu.cn (N.W.); khe@sjtu.edu.cn (K.H.)

**Keywords:** insulin pathway, IRTKS, SHIP2, PI(3,4,5)P_3_

## Abstract

Insulin signaling is mediated by a highly integrated network that controls glucose metabolism, protein synthesis, cell growth, and differentiation. Our previous work indicates that the insulin receptor tyrosine kinase substrate (IRTKS), also known as BAI1-associated protein 2-like 1 (BAIAP2L1), is a novel regulator of insulin network, but the mechanism has not been fully studied. In this work we reveal that IRTKS co-localizes with Src homology (SH2) containing inositol polyphosphate 5-phosphatase-2 (SHIP2), and the SH3 domain of IRTKS directly binds to SHIP2’s catalytic domain INPP5c. IRTKS suppresses SHIP2 phosphatase to convert phosphatidylinositol 3,4,5-triphosphate (PI(3,4,5)P_3_, PIP3) to phosphatidylinositol (3,4) bisphosphate (PI(3,4)P_2_). IRTKS-knockout significantly increases PI(3,4)P_2_ level and decreases cellular PI(3,4,5)P_3_ content. Interestingly, the interaction between IRTKS and SHIP2 is dynamically regulated by insulin, which feeds back and affects the tyrosine phosphorylation of IRTKS. Furthermore, IRTKS overexpression elevates PIP3, activates the AKT–mTOR signaling pathway, and increases cell proliferation. Thereby, IRTKS not only associates with insulin receptors to activate PI3K but also interacts with SHIP2 to suppress its activity, leading to PIP3 accumulation and the activation of the AKT–mTOR signaling pathway to modulate cell proliferation.

## 1. Introduction

We previously isolated insulin receptor tyrosine kinase substrate (IRTKS), also known as BAI1-associated protein 2-like 1 (BAIAP2L1), from human endocrine organs (GenBank accession number: AF119666) [1]. It encodes a member of the IRSp53/MIM (missing-in-metastasis) homology domain family. Other members of this family have been studied well and they play an important role in the formation of plasma membrane protrusions [2]. IRSp53 is a founding member of the IRSp53/MIM homology domain family, which shares 40% identical sequence with IRTKS and was originally identified as a tyrosine phosphorylation substrate of insulin receptor (IR) [3,4]. IRTKS is frequently overexpressed in human hepatocellular carcinoma (HCC) and gastric cancer, and promotes cell proliferation [5]. Recently we showed that IRTKS is an adaptor of insulin receptor that modulates the insulin-receptor substrate (IRS)–PI3K–AKT signaling pathway via regulation of the phosphorylation of IR and IRS, whilst IRTKS deficiency causes insulin resistance in mice [6].

Although IRTKS has been proved to regulate the insulin signaling pathway, the detailed mechanism is unclear. Through a two-hybrid yeast screening, we identified one potential partner of IRTKS, Src homology (SH2) containing inositol polyphosphate 5-phosphatase-2 (SHIP2), which specifically hydrolyzes the 5-phosphate of phosphatidylinositol 3,4,5-triphosphate (PI(3,4,5)P_3_, PIP3) to produce PI(3,4)P_2_. SHIP2 has been demonstrated to inhibit AKT activation in HepG2 cells [7], while a low expression of SHIP2 enhances the PI3K–AKT signaling to promote cell proliferation and tumorigenesis in gastric cancer [8]. Inhibition of SHIP2 activity prevents cell migration and metastasis in breast cancer cells [9]. These studies indicate that SHIP2 may play an important role in regulating PI3K–AKT signaling transduction.

In the present work, we demonstrate that IRTKS interacts with SHIP2 and suppresses its phosphoinositide phosphatase activity, leading to PIP3 accumulation, which further provokes the insulin downstream signaling and increases cell proliferation. This study provides a new insight into the IR–PI3K–AKT network regulation.

## 2. Results

### 2.1. IRTKS Interacts with SHIP2

**Figure 1:**
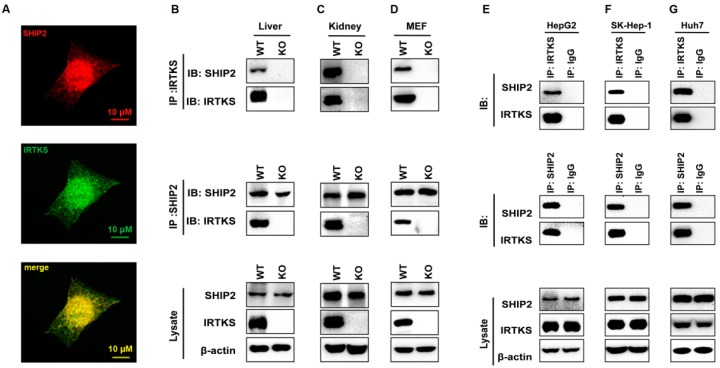
Insulin receptor tyrosine kinase substrate (IRTKS) interacts with Src homology (SH2) containing inositol polyphosphate 5-phosphatase-2 (SHIP2) in vivo. (**A**) Co-localization of IRTKS and SHIP2. HepG2 cells were immunostained with anti-IRTKS antibody (1:50) and anti-SHIP2 antibody (1:50) followed by Alexa-488 conjugated anti-rabbit antibody (1:1000) and Alexa-647 conjugated anti-mouse antibody (1:1000), respectively. Images were taken with a confocal microscope (Nikon & A1Si). (**B**–**G**) The interaction between IRTKS and SHIP2 was detected by co-immunoprecipitation (co-IP) with anti-IRTKS and anti-SHIP2 antibodies in liver, kidney, and mouse embryonic fibroblasts (MEFs) from wild-type (WT) and IRTKS-knockout (KO) mice, as well as in HepG2, SK-Hep-1, and Huh7 cells. The Immunoglobulin G (IgG) group was the negative control.

To validate the interaction between IRTKS and SHIP2, we first tried immunofluorescence staining with anti-IRTKS and anti-SHIP2 antibodies and observed that IRTKS and SHIP2 were co-localized in the plasma membrane, cytoplasm, and nucleus (Figure 1A). This result suggests that IRTKS could physically associate with SHIP2. To double-check the interaction, we performed a co-immunoprecipitation (Co-IP) assay with the lysates of mouse embryonic fibroblasts (MEFs) as well as liver and kidney tissues isolated from IRTKS-knockout (KO) mice. As the data shown in Figure 1B–D, IRTKS and SHIP2 can reciprocally immunoprecipitate each other with anti-SHIP2 and anti-IRTKS antibodies, respectively. The interaction between IRTKS and SHIP2 was also observed in human liver cancer cell lines HepG2, SK-Hep-1, and Huh7 (Figure 1E–G). These results confirm that IRTKS interacts with SHIP2.

### 2.2. The Interaction between IRTKS and SHIP2 Is Mediated by the SH3 and IPP5c Domains

**Figure 2:**
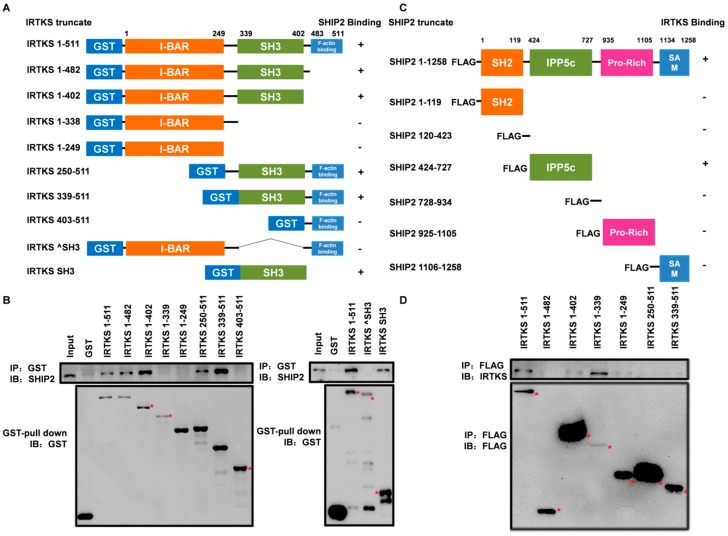
The SH3 domain of IRTKS binds to the inositol polyphosphate-5-phosphatase domain (IPP5c) of SHIP2. (**A**) A schematic diagram of glutathione S-transferase (GST)-tagged IRTKS constructs encoding its full length and truncated proteins. (**B**) GST-tagged IRTKS fusion proteins purified from *Escherichia coli* (*E. coli*) (BL 21) were added to the HEK293T cell lysates to pull down SHIP2. (**C**) A schematic diagram of Flag-tagged SHIP2 constructs encoding its full length and truncated proteins. (**D**) The Flag-tagged SHIP2 fusion proteins overexpressed in HEK293T were pulled down by endogenous IRTKS. Red asterisks indicate the fusion proteins with correct molecular weight.

To unveil which domains of IRTKS and SHIP2 are responsible for their interaction, we first made a series of constructs encoding different IRTKS truncates fused to glutathione S-transferase (GST) (Figure 2A). We then performed the GST pull-down assays with IRTKS fusion proteins purified from *Escherichia coli* (*E. coli*) (BL 21) and endogenous SHIP2. The results showed that the GST-tagged IRTKS proteins containing SH3 domain can pull down SHIP2 in HEK293T cells, whereas the fragments lacking the SH3 domain failed to pull down SHIP2 (Figure 2A,B), indicating that the SH3 domain is crucial for the binding of IRTKS to SHIP2.

We also constructed a series of plasmids encoding different functional domains of SHIP2 and purified their Flag-tagged proteins (Figure 2C). The pull-down assay with Flag-tagged full-length or truncated SHIP2 proteins confirmed the interaction between IRTKS and SHIP2 (Figure 2C,D). Interestingly, the fragment of SHIP2 (residues 424–727) with the IPP5c domain responsible for PtdIns phosphatase can pull down IRTKS; in contrast, other fragments containing SH2, SAM and/or proline-rich domains cannot pull down IRTKS (Figure 2D), proving that the IPP5c phosphatase domain mediates the binding of SHIP2 to IRTKS.

### 2.3. IRTKS Inhibits SHIP2 Enzyme Activity

**Figure 3:**
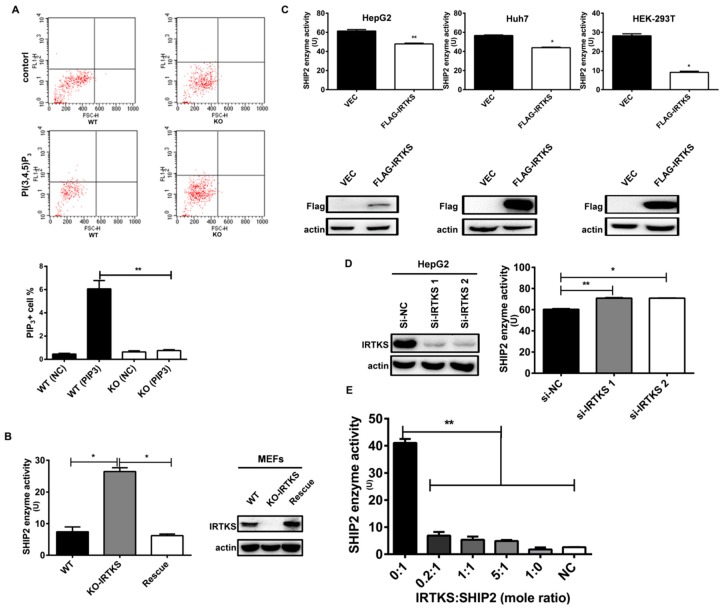
IRTKS suppresses SHIP2 phosphatase activity. (**A**) The PI(3,4,5)P_3_ in WT and IRTKS KO MEFs stimulated by insulin for 15 min was labeled by anti-PI(3,4,5)P_3_ antibody and assessed by a flow cytometer. Control cells were incubated with bovine serum albumin (BSA); (**B**) The SHIP2 activity was significantly increased in IRTKS KO MEFs, while the ectopic IRTKS rescue decreased SHIP2 activity. Here SHIP2 proteins immunoprecipitated with anti-SHIP2 antibody in WT, IRTKS KO, and the IRTKS-rescued MEFs were used to determine its phosphatase activity via malachite green phosphatase assay; (**C**) Over-expression of IRTKS attenuated SHIP2 phosphatase activity in HepG2, Huh7, and HEK293T cells; (**D**) Knocking down IRTKS promoted SHIP2 phosphatase activity in HepG2 cells. (**E**) The IRTKS/SHIP2 ratio affected SHIP2 phosphatase activity. Different amounts of Flag–IRTKS and Flag–SHIP2 proteins purified from HEK293T cells were added in the malachite green phosphatase assay. Fluorescence Activated Cell Sorter (FACS) assay and phosphatase assay were performed in triplicates. The data are shown as mean ± SD. * *p* < 0.05, ** *p* < 0.01 (Student’s *t*-test).

As a 5′-phosphotase of PtdIns, SHIP2 regulates the level of PIP_3_, a well-known lipid second messenger involved in various cellular signaling pathways including the Insulin–PI3K–AKT signaling pathway [10]. In the present work, the interaction between IRTKS and SHIP2 inspired us to investigate whether IRTKS can affect SHIP2 activity and subsequent PIP3 level. We first measured PIP_3_ level in wild-type (WT) and IRTKS-KO MEFs under insulin stimulation. The results showed that the level of PIP_3_ was significantly decreased in IRTKS-KO MEFs under insulin stimulation, as compared to that of WT MEFs (Figure 3A), implying that IRTKS deficiency could promote SHIP2 to dephosphorylate PIP3 and convert it to PtdIns(3,4)P_2_. This suggests that IRTKS function as an inhibitory factor of SHIP2 enzyme.

To validate whether IRTKS affects the activity of SHIP2, we performed the SHIP2 phosphatase assay with or without IRTKS. As expected, SHIP2 showed a significantly higher enzyme activity in IRTKS-KO MEFs than that of WT MEFs, and the ectopic IRTKS rescue can suppresses the SHIP2 phosphatase activity (Figure 3B), indicating that IRTKS can inhibit SHIP2 enzyme activity in mouse cells.

Subsequently, we overexpressed IRTKS in human cell lines, including HepG2, Huh7, and HEK293T cells. The phosphatase activity of SHIP2 purified from the lysates of IRTKS-overexpressed cells was significantly lower than that of control cell lysates (Figure 3C). Conversely, IRTKS knockdown with the synthesized small interference RNAs (siRNA) increased the enzyme activity of SHIP2 in HepG2 cells, compared to the unrelated siRNA (Figure 3D).

Furthermore, we try to address whether IRTKS can directly inhibit the enzyme activity of SHIP2 through conducting in vitro phosphatase assay with the recombinant SHIP2 and IRTKS proteins purified from HEK293T cells. Interestingly, the SHIP2 alone showed very high phosphatase activity, while its activity was significantly decreased when the recombinant IRTKS proteins were added (Figure 3E). Our work showed that IRTKS can directly inhibit SHIP2 enzyme activity.

### 2.4. Interaction between IRTKS and SHIP2 Is Dynamically Regulated by Insulin

As known, the proteins interaction and their modifications involved in insulin signaling transduction are usually dynamically regulated during insulin stimulation. Our previous work showed that the tyrosine phosphorylation level of IRTKS reached to its peak in 15–30 min after insulin stimulation, and then decreased, exhibiting a similar dynamic-pattern to insulin in mouse liver [6].

**Figure 4:**
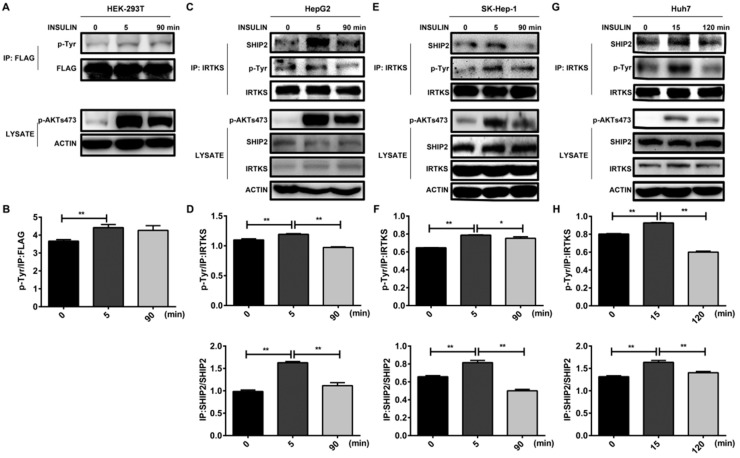
IRTKS dynamically interacts with SHIP2 under insulin stimulation. (**A**,**B**) Insulin stimulation increased the tyrosine phosphorylation of IRTKS. Flag–IRTKS fusion protein was expressed in HEK293T cells following insulin stimulation. After immunoprecipitation with FLAG M2 beads, Western blotting was performed to detect the tyrosine phosphorylation of IRTKS. (**C**–**G**) Insulin stimulation enhanced the endogenous IRTKS–SHIP2 interaction. The SHIP2 was immunoprecipitated by anti-IRTKS antibody in Huh7, SK-Hep-1, and HepG2 cells after insulin treatment (**C**,**E**,**G**). The tyrosine phosphorylation of IRTKS and the immunoprecipitated SHIP2 by anti-IRTKS antibody (IP: SHIP2) were quantitated based on the intensity of their bands, and then normalized according to total IRTKS and SHIP2 with three replicates, respectively (**D**,**F**,**H**). The data are shown as mean ± SD. * *p* < 0.05, ** *p* < 0.01 (Student’s *t*-test).

In this work, to answer whether the interaction between IRTKS and SHIP2 is dynamically regulated by insulin. We first assessed total tyrosine phosphorylation level of IRTKS during insulin stimulation, showing that the tyrosine phosphorylation level of IRTKS was increased in 5 min and attenuated in 90 min after insulin stimulation in human HEK293T cells (Figure 4A) as well as liver cancer cell lines SK-Hep-1, Huh7, and HepG2 (Figure 4B–G). The dynamic change of IRTKS phosphorylation level is consistent with the pattern of AKT phosphorylation on serine 473, a well-known response to insulin stimulation (Figure 4A). Then, we performed co-immunoprecipitation and found more SHIP2 was immunoprecipitated by IRTKS in 5–15 min after insulin stimulation when IRTKS tyrosine phosphorylation reached to its peak, whilst the immunoprecipitation of SHIP2 by IRTKS was attenuated in 90–120 min when IRTKS tyrosine phosphorylation decayed (Figure 4B–G), indicating that the interaction between IRTKS and SHIP2 could be positively correlated with the tyrosine phosphorylation level of IRTKS as well as the insulin downstream AKT phosphorylation. This might due to the fact that IRTKS interacts with SHIP2 and inhibits its enzyme activity, which leads to PIP3 accumulation and AKT activation.

### 2.5. IRTKS Promotes Insulin Signaling and Cell Proliferation Partially Due to Inhibiting SHIP2 Activity

**Figure 5:**
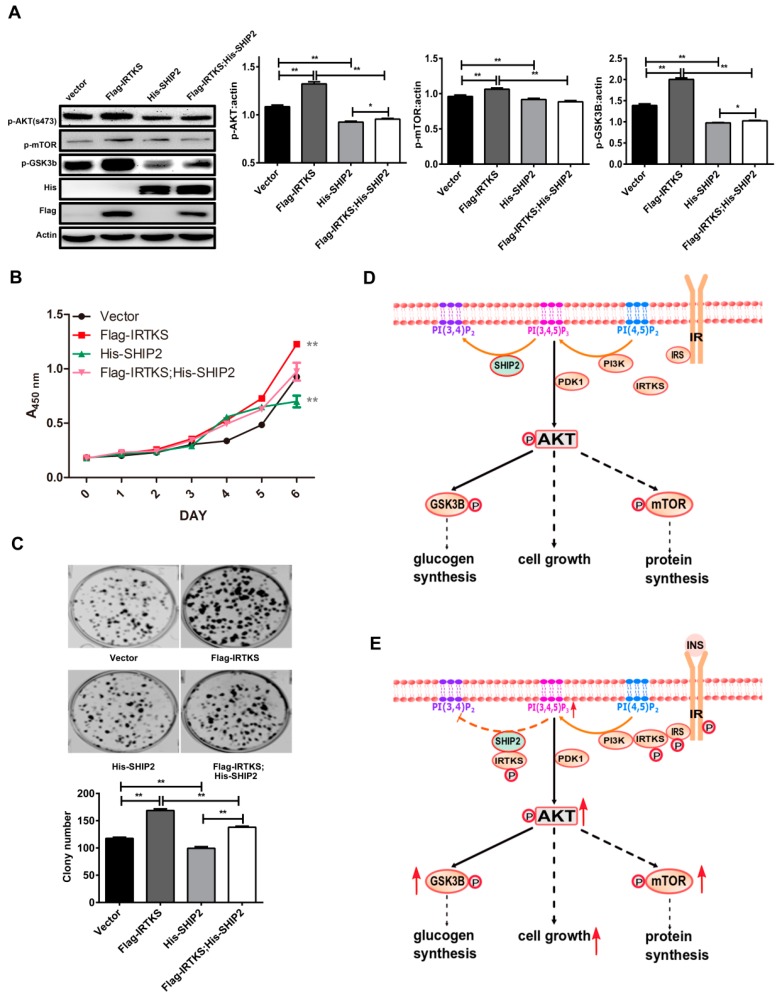
IRTKS promotes insulin signal transduction and cell proliferation partially due to inhibiting SHIP2 activity. (**A**) SK-Hep-1 cells transfected with Flag–IRTKS and/or His–SHIP2 were stimulated with insulin for 15 min. The phospho-AKT(S473) (p-AKT), phospho-mammalian target of rapamycin (S2448) (p-mTOR), and phospho-glycogen synthase kinase-3 beta (S9) (p-GSK3β) were detected by Western blotting assay and were quantitated with three replicates. The data are shown as mean ± SD. * *p* < 0.05, ** *p* < 0.01 (Student’s *t*-test). (**B**) The proliferation curve of SK-Hep-1 cells overexpressing Flag–IRTKS or His–SHIP2 was monitored by cell counting kit-8 (CCK8) at the indicated times. (**C**) Colony formation of SK–Hep-1 cells overexpressing Flag–IRTKS or His–SHIP2 was determined by Coomassie blue. (**D**,**E**) Schematic diagrams of molecular mechanism involved in the IRTKS enhanced insulin signaling transduction through inhibiting SHIP2 enzyme activity.

To further explore the function of IRTKS and SHIP2 interaction, we monitored the downstream molecules of insulin signaling pathway (Figure 5A). In consistence with our previous work, ectopic IRTKS overexpression significantly increased the phosphorylation of AKT, mammalian target of rapamycin (mTOR), and glycogen synthase kinase-3 beta (GSK3β). Ectopic SHIP2 overexpression suppressed the phosphorylation of AKT, mTOR, and GSK3β, which was partially reversed by ectopic IRTKS overexpression (Figure 5A). These results could be partially ascribed to IRTKS directly binding to SHIP2 and inhibiting its activity, and therefore promoting the AKT–mTOR signaling pathway.

As known, insulin signaling transduction could promote cell proliferation [11]. With insulin stimulation, IRTKS is phosphorylated by IR and then enhances PI3K activity, increasing the phosphorylation of PIP2 to produce PI(3,4,5)P3. The phosphorylated IRTKS simultaneously attenuates SHIP2 activity, inhibiting the de-phosphorylation of PI(3,4,5)P3. Both result in the increase of PI(3,4,5)P3 to further trigger phosphoinositide-dependent protein kinase (PDK), AKT, and mTOR activation. To investigate whether IRTKS affects cell proliferation through interaction with SHIP2, we monitored the cell growth curve and colony formation. As expected, IRTKS overexpression promoted cell proliferation and colony formation, whilst SHIP2 overexpression inhibited these cellular phenotypes (Figure 5B,C). The ectopic IRTKS expression partially relieved the inhibitory effect of SHIP2 on the insulin downstream molecules and cell proliferation (Figure 5A). These results suggested that IRTKS promotes insulin signaling and cell proliferation, partially due to inhibiting SHIP2 activity.

## 3. Discussion

In the present work we reveal that IRTKS directly interacts with SHIP2 and inhibits its phosphatase activity. IRTKS mainly contains the N-terminal I-BAR domain, SH3 domain, and the F-actin binding domain. The I-BAR domain is a common structure of I-BAR superfamily members, and can induce plasma membrane protrusion formation [12]. The SH3 domain, known as a protein recognition module, could serve as a docking harbor [13]. Here, we found that the SH3 domain mediates the binding of IRTKS to SHIP2.

The SH2 domain of SHIP2 is usually considered as a harbor platform for interacting with other proteins [14]. IPP5c is a vital part of SHIP2 phosphatase activity which hydrolyzes the 5-position of PI(3,4,5)P_3_ to produce PI(3,4)P_2_ [14]. The inositol 5-phosphatases activity of SHIP2 directly affects the level of PI(3,4,5)P_3_, a key second lipid messenger for insulin signaling transduction. Interestingly, our results showed that the IPP5c domain is responsible for the binding of SHIP2 to IRTKS, supporting that IRTKS binding to SHIP2 can inhibit its activity.

It has been known that SHIP2 phosphatase activity can usually be modulated through phosphorylation and interaction with specific interactors, which could be influenced by feed back to SHIP2 [15]. In the present work, we confirmed that IRTKS directly inhibits SHIP2 enzyme activity in MEFs and human liver cells. According to a published study [16], SHIP2 possesses a flexible loop (Gly676 to Asn684), which is capable of folding over the enzyme active site once SHIP2 binds its ligand. Here we observe that the IPP5c domain, also containing a flexible loop, is responsible for the binding of SHIP2 to IRTKS. Our data showed that not only does IRTKS directly bind to SHIP2 and suppress its phosphoinositide phosphatase activity, but also SHIP2 feeds back and affects IRTKS’s phosphorylation.

Insulin signaling is mediated by a highly integrated network that controls glucose metabolism, protein synthesis, cell growth, and differentiation. Insulin provokes IR phosphorylation and triggers its downstream PI3K–AKT–mTOR signaling pathway activation. SHIP2 is a potent negative regulator of PI3K–AKT signaling in vivo. Loss of SHIP2 increases the sensitivity to insulin, resulting in the AKT phosphorylation in MDB-MA-231 cells [9]. IRTKS, as an adaptor protein, has recently been found to bind to IR, leading to enhanced insulin signaling, whereas IRTKS deficiency leads to insulin resistance in mice [5]. Considering that the human liver is the major organ for metabolism regulated by insulin signals, we employed SK-Hep-1, Huh7, and HepG2 cells derived from liver cancer in this study. In the current work, our data further support our previous findings that IRTKS enhances insulin signaling transduction. The data also infer a notion that IRTKS promotes the generation of PI(3,4,5)P_3_, an active signal molecule for insulin signaling pathway, through two ways: one is that IRTKS enhances the phosphorylation of IR, leading PI3K activation to convert PI(4,5)P_2_ to PI(3,4,5)P_3_; the other is that IRTKS directly binds to SHIP2 and suppresses the conversion of PI(3,4,5)P_3_ to PI(3,4)P_2_ (Figure 5D,E). The finding provides a new insight to understand the regulation of the insulin signaling pathway through balancing phosphoinositides.

The Akt–mTOR axis is directly under the control of PIP3 level, which is regulated by PI3K, phosphatase and tensin homologue deleted on chromosome ten (PTEN), and SHIP2. As we know, PI3K phosphorylates PI(4,5)P2 to produce PIP3, while SHIP2 and PTEN dephosphorylate PIP3 to produce PI(3,4)P2 and PI(4,5)P2, respectively. PI3K is a complex which includes p85α and p85β regulatory subunits and p110 catalytic subunit, and its activity is regulated by p85 subunits and other regulators, such as PI3 kinase enhancer (PIKE) [17]. Our work showed that IRTKS interacted with SHIP2, and overexpression of IRTKS repressed SHIP2 activity to increase PIP3, while knockout of IRTKS enhanced SHIP2 activity and decreased PIP3. The SHIP2-governed PIP2 and PIP3 equilibrium is regulated by IRTKS, which can be integrated to the whole network of PI3K-, PTEN-, and SHIP2-governed PIP2 and PIP3 equilibrium. IRTKS overexpression evokes the AKT and mTOR downstream signaling pathway and increases cell proliferation. Higher IRTKS expression and lower SHIP2 expression were detected in some tumors, both leading to PIP3 increase and AKT–mTOR activation. The over-activation of the PI3K–AKT–mTOR is very common in cancer cells and plays an important role in tumorigenesis [18]. Taken together, we might make a hypothesis that IRTKS promotes tumorigenesis through suppressing SHIP2 activity and enhancing PI3K to increase the PIP3 level that activates the AKT–mTOR pathway. Therefore, it is possibility that the IRTKS–SHIP2 interaction could be useful as a possible therapeutic target. The SHIP2 enzyme activation could be restored through disrupting the bind of IRTKS to SHIP2, where targeting SH3 domain of IRTKS or the interface between SH3 domain and IPP5c domain of SHIP2 could be appropriate for drug screening using chemical libraries or bioinformatic tools based on their crystal structure.

Both IRTKS and SHIP2 co-immunoprecipitation assay showed the interaction between IRTKS and SHIP2 was dynamically regulated by insulin, which was consistent with the AKT phosphorylation. The function of this dynamic regulation has not been fully studied in the current work and could be an interesting issue for future study. We only measured the tyrosine phosphorylation of IRTKS and did not assess the serine and threonine phosphorylation of IRTKS. Whether the tyrosine phosphorylation and the serine/threonine phosphorylation of IRTKS enhance or suppress the IRTKS–SHIP2 interaction should be addressed in the future work.

## 4. Materials and Methods

### 4.1. Cells and Reagents

HEK293T, SK-Hep-1, Huh7, and HepG2 cells were purchased from the Institute of Biochemistry and Cell Biology, Shanghai Institutes for Biological Sciences, Chinese Academy of Sciences. The wild type and IRTKS knockout mouse embryonic fibroblasts (MEFs) were isolated from E12.5 embryos. All these cells were routinely maintained in Dulbecco’s modified Eagle’s medium supplemented with 10% fetal bovine serum, 100 units/mL of penicillin, and 100 g/mL of streptomycin. The cells were serum-starved for 16 h followed insulin stimulation for the indicated time. Anti-IRTKS rabbit polyclonal antibody (1:300 for Western blot) was produced in the laboratory [19]. Anti-Flag (Cat: sc-166355) and anti-SHIP2 antibodies (1:200 for western blot, Cat: sc-166641) were from Santa Cruz Biotechnology (Dallas, TX, USA). Anti-phospho-AKT (Ser473) (1:1000 for western blot, Cat: 4060), anti-AKT (1:3000 for western blot, Cat: 4685), anti-phospho-GSK3β (Ser9) (1:1000 for western blot, Cat: 9323), anti-phospho-mTOR (ser2448) (1:1000 for western blot, Cat: 5536), and anti-phosphorylated Tyr antibodies (1:300 for western blot, Cat: 9411) were obtained from Cell Signaling Technology (Danvers, MA, USA). Anti-actin (1:5000 for western blot, Cat: A5441) antibody and other reagents were purchased from Sigma Aldrich (St. Louis, MO, USA). All animal experiments were approved by the Animal Use and Care Committee of Shanghai Jiao Tong University. The animal protocol number is A2016008, and it was approved on 15 April 2016.

### 4.2. siRNA, Plasmids, and Transient Transfection

Two siRNAs against IRTKS were designed and chemically synthesized by Shanghai GenePharma Co. Ltd (Shanghai, China). siRNA-1, sense, 5′-CCA GUC CCU UGA UCG AUA UTT-3′ and anti-sense, 5′-AUA UCG AUC AAG GGA CUG GTA-3; siRNA-2, sense, 5′-GCU UAA GCA AAU CAU GCU UTT-3′ and anti-sense, 5′-AAG CAU GAU UUG CUU AAG CAG-3′.

Full-length IRTKS and fragments were amplified from human cDNA by PCR using Q5 polymerase and were sub-cloned into pGEX-4T-1 vectors (GE Healthcare Life Sciences (Pittsburgh, PA, USA)). The human SHIP2 was purchased from Youbio Corporation (Changsha, China) and its fragments were sub-cloned into pCDNA3.1 (+) vectors. Flag–IRTKS, Flag–SHIP2, and His–SHIP2 were amplified by PCR using Q5 polymerase, and then sub-cloned into pCDH–CMV–MCS–EF1-copGFP (System Bioscience, Palo Alto, CA, USA).

The plasmid or siRNA was transfect to cells using Lipofectamine 2000 (Invitrogen, Grand Island, NY, USA) according the manufacturer’s instructions,

### 4.3. Immunoprecipitation Assay

The cell lysates (1 mg total protein) from tissues or cultured cells were incubated with the indicated antibodies (1 μg) at 4 °C overnight, and then incubated with protein-G beads at 4 °C for another 4 h. The beads were washed four times and analyzed by immunoblotting assay.

### 4.4. GST Pull-Down Assay

The GST-tagged IRTKS recombinant proteins were purified from *E. coli* by glutathione-sepharose beads. GST-tagged IRTKS fusion proteins bound to beads were incubated with HEK293T cells lysate overnight at 4 °C. After washing four times with lysis buffer, the samples were analyzed by Western blot.

### 4.5. Western Blot Assay

After boiling 5 min in 1× loading buffer, the samples were separated by SDS-polyacrylamide gel electrophoresis and transferred to nitrocellulose membranes (Millipore, Burlington, MA, USA). The membranes were incubated with specific primary antibodies, secondary antibodies and then visualized by ProteinSimple system (ProteinSimple, San Jose, CA, USA).

### 4.6. SHIP2 Enzyme Activity Assay

The phosphatase activity of SHIP2 was measured by malachite green phosphatase assay kit (Echelon Bioscience, Salt Lake City, UT, USA) according to manufactory protocol. Briefly, SHIP2 was immunoprecipitated from the lysate of HEK293T cells in which the IRTKS were overexpressed or knocked down. Beads were washed three times with lysis buffer and twice with malachite green assay buffer (25 mM Tris-Cl, pH 7.4, 140 mM NaCl, 2.7 mM KCl). Then, 3 nM PIP(3,4,5)P_3_ (Echelon Bioscience) substrate was added to 25 μL of reaction buffer containing purified SHIP2 protein and different amounts of recombinant Flag–IRTKS protein. Samples were incubated at room temperature (RT) for 60 min, followed by addition of 100 μL of malachite green stop solution at RT. Absorbance at 630 nm was measured to calculate free phosphate concentration which was used to evaluate enzyme activity.

### 4.7. PIP3 Determinate Assay

Cells were collected and washed three times with cold PBS, then blocked with BSA for 30 min on ice, and incubated with anti-PIP3 antibody (Echelon Bioscience) for 1 h. After being washed three times with PBS, the cells were incubated with fluoresceine isothiocyanate (FITC)-second antibody for another 1 h, and washed four times with PBS. Then the cells were assessed on a Fluorescence Activated Cell Sorter (FACS) Calibur flow cytometer.

### 4.8. Cell Proliferation Curve

Cells were seeded in 96-well plates with 2000 cells/well in triplicate. After culture for the indicated time, the cell viability was monitored with a Cell Counting Kit-8 (CCK-8, Dojindo Laboratories, Kumamoto, Japan) according to the manufacturer’s instructions.

### 4.9. Cell Colony Formation Assay

Following our previous study [20], cells were seeded in 6-well plate with 3000–5000 cells/well in triplicate. After 2–3 weeks, clones were stained with Coomassie Blue and counted.

### 4.10. Quantification and Statistical Analyses

All experiments were carried out at least three times. Western blot was quantified with the Quantity One software version 4.6.9 (Bio-Rad). One- and two-way ANOVA with Boneferonni or Tuckey post hoc and *t*-tests were performed with GraphPad Prism version 5 (GraphPad Software, San Diego, CA, USA).
